# *Serratia marcescens*: A Rare Cause of Recurrent Implantable Cardioverter Defibrillator Site Infection

**DOI:** 10.1155/2015/641297

**Published:** 2015-10-29

**Authors:** S. Hawkey, AM. Choy

**Affiliations:** Division of Cardiovascular & Diabetes Medicine, Ninewells Hospital & Medical School, University of Dundee, Dundee DD1 9SY, UK

## Abstract

We present a unique case of a patient who experienced recurrent infections of his implantable cardioverter defibrillator (ICD) site with the bacterium *Serratia marcescens*. This report highlights the virulence of this bacterium, its resistance to antibiotic therapy, and its ability to remain latent for prolonged periods between episodes of sepsis. It also demonstrates the merits of reimplanting devices at different sites in the context of *Serratia marcescens* infection.

## 1. Introduction

As the burden of cardiovascular disease continues to rise, more patients are being managed with implantable electronic devices (ICDs) in clinical practice [1]. However, the use of increasingly complex devices, combined with an ageing population with multiple comorbidities, has meant that device related infection has also risen [2].* Staphylococcal* species are the most frequent cause of device infections, accounting for approximately 70% to 90% of cases [3–5]. We present a salutary case of recurrent and latent infections of an ICD device site with the bacterium* Serratia marcescens*.

## 2. Case Report

A 73-year-old nondiabetic male with idiopathic cardiomyopathy underwent uncomplicated ICD implantation in the left subclavicular area for sustained ventricular tachycardia (VT). Two weeks later, he presented with septicaemia and infection with* Serratia marcescens* at the ICD pocket site. Complete extraction of the device and a six-week course of co-trimoxazole resulted in clinical improvement with normalisation of infection markers and good healing of the infected site. Eight weeks later, another implant was attempted on the same side but was aborted after skin incision, as venous access could not be achieved. Two weeks following the procedure, the patient developed an abscess in the site of the attempted implant that grew* Serratia marcescens*. Drainage of the wound followed by a prolonged course of Trimethoprim resulted in complete clinical recovery.

Thereafter, the patient's recurrent VT was managed pharmacologically with amiodarone. The patient remained free of signs and symptoms of infection for eleven months when another ICD was considered for repeat implantation on the left side. Although this was the side of previous infection, implantation on the left was preferred as there were concerns regarding high defibrillation threshold from a right sided ICD implant, given the underlying degree of cardiomegaly. The previous wound site was also healthy and the patient had been free of infection for eleven months. An ICD was successfully implanted under aseptic technique with perioperative intravenous prophylactic flucloxacillin as per local protocol. Three weeks after procedure, the patient presented with septicaemia, with* Serratia marcescens* grown from blood cultures. Although serotyping was not available, the identical antibiotic sensitivities to the previous infections suggested the same strain. There were, however, no signs or symptoms of infection in the ICD pocket. Transesophageal echocardiography did not reveal vegetations on the valves or the ICD leads. Urinary investigations did not show colonisation with* Serratia marcescens*. Immunological investigations did not indicate an immunocompromised state. The patient responded well clinically to a two-week course of Ertapenem, which was followed by three months of Trimethoprim to eradicate any latent infection. As the ICD pocket did not appear to be infected, the device was not removed during this admission.

Following discharge, the patient remained well for a further eight months until he presented to hospital with pyrexia of unknown origin (PUO) with rigors and elevated C-reactive protein. Infection screen was negative, as were other tests for noninfectious causes of PUO. Although the ICD pocket did not appear infected clinically, no other cause for sepsis was identified, and exploration of the ICD pocket was undertaken. Findings during pocket exploration did not suggest infection; however, cultures from the pocket tissue grew* Serratia marcescens*. As a result, the ICD system was completely explanted with excision of the capsule and a two-month course of chloramphenicol was given. Two months later, the patient underwent successful VT ablation and remained free of arrhythmia for a further 25 months until he required a pacemaker for bradycardia. The device was implanted on the opposite (right) side of the chest and the patient has remained well for over three years ever since with no recurrence of* Serratia marcescens* infection.

## 3. Discussion

*Serratia marcescens* is a gram negative bacterium that is part of the Enterobacteriaceae family. Rarely seen in the community, it is a pathogen that is becoming increasingly associated with hospital acquired infection [6], with data from 2009 to 2011 showing that* Serratia* accounted for an average of 6.5% of all gram negative infections in intensive care units in USA and Europe [7]. As an opportunistic pathogen,* Serratia marcescens* most commonly affects patients with immunodeficiency disorders, as well as those receiving broad spectrum antibiotic therapy or who have indwelling urinary or intravenous catheters [8]; however, our patient did not have any of these predisposing conditions.

To our knowledge, this is the first reported case of a nonimmunocompromised patient experiencing recurrent ICD infections with* Serratia marcescens*, appearing to remain latent for extensive periods after prolonged and seemingly successful courses of antibiotic therapy between episodes of sepsis. This ability to remain latent was previously reported in an immunocompromised diabetic patient, who developed an infective pericardial effusion with* Serratia marcescens* after cardiac transplantation. Despite successful antibiotic treatment and clinical recovery, the patient developed septicaemia 15 years later secondary to sternal osteitis caused by the same strain of* Serratia marcescens* [9].

Although* Serratia marcescens* has been previously associated with low pathogenicity, virulence factors have been identified such as production of cytolysin, which is thought to promote cell cytotoxicity and release of inflammatory mediators [10]. Additional pathogenic factors associated with* Serratia marcescens* include the production of biofilms and AmpC beta-lactamase, which are thought to have contributed to its increasing ability to evade host defences and resist antibiotic therapy [11, 12]. Studies have shown that* Serratia marcescens* is resistant to a wide range of antibiotic classes including penicillins, cephalosporins, and macrolides [13]. Although aminoglycosides, fluoroquinolones, and third generation cephalosporins have traditionally been effective, there is now increasing evidence of resistance to these drugs [14, 15]. One class of antibiotics that are often the agent of choice is carbapenems, which remain effective against bacteria despite beta-lactamase production. However, there are now emerging cases of carbapenemase-mediated resistance in* Serratia marcescens* within the literature [16, 17]. Although these are isolated outbreaks, the developing pattern of multidrug resistance seen in other gram negative bacteria means this is likely to become an increasing problem, rendering current treatment options severely restricted.

This capacity of* Serratia marcescens* to resist antibiotic therapy in vivo in spite of the in vitro sensitivity was exemplified by our case in that multiple courses of antibiotics failed to eradicate the bacterium from its original site. The capability of this bacterium to “hide itself” from antibiotic therapy and remain dormant suggests that in the context of device infection with* Serratia marcescens*, removing the device and reimplanting it at a different site are critical in order to prevent disease recurrence. As occurred in our patient, device implants infected with* Serratia marcescens* can trigger sepsis, multiple hospitalisations, device reimplantation, and significant morbidity, thus highlighting the need for awareness of this bacterium and its capacity to infect device sites and remain latent for prolonged asymptomatic periods despite seemingly effective antimicrobial therapy. This case also demonstrates the value of reimplanting devices at different sites in the context of recurrent infection, even if there are no signs of infection in the affected area.
